# Generation of mutation hotspots in ageing bacterial colonies

**DOI:** 10.1038/s41598-016-0005-4

**Published:** 2016-12-05

**Authors:** Agnieszka Sekowska, Sofie Wendel, Emil C. Fischer, Morten H. H. Nørholm, Antoine Danchin

**Affiliations:** 1 Institute of Cardiometabolism and Nutrition, CHU Pitié-Salpêtrière, 47, Boulevard de l’Hôpital, 75013 Paris, France; 2 Novo Nordisk Foundation Center for Biosustainability, Technical University of Denmark, Kogle Alle 6, DK-2970 Hørsholm Denmark

## Abstract

How do ageing bacterial colonies generate adaptive mutants? Over a period of two months, we isolated on ageing colonies outgrowing mutants able to use a new carbon source, and sequenced their genomes. This allowed us to uncover exquisite details on the molecular mechanism behind their adaptation: most mutations were located in just a few hotspots in the genome, and over time, mutations increasingly were consistent with the involvement of 8-oxo-guanosine, formed exclusively on the transcribed strand. This work provides strong support for retromutagenesis as a general process creating adaptive mutations during ageing.

## Introduction

Bacteria constitute a precious biological model system for studying the molecular details of ageing and evolution. Bacterial cells defective in the MutY enzyme, responsible for removing adenine nucleotides pairing with the 8-oxo oxidized variant of guanosine (8-oxo-G), exhibit a dramatic increase in the number of adaptive mutants and Bridges proposed a model to explain this phenomenon that was later termed retromutagenesis^1, 2^. In this model, the process of transcription opens up the DNA double helix, enhancing the probability of mutation in the transcribed strand, but only mutations on the transcribed strand are transferred to mRNA and translate into mutant proteins that explore novel activities. Subsequently, the activity of the mutant protein may enable the cell to replicate and thereby to fix the initial adaptive mutation on both DNA strands. In agreement with the retromutagenesis model, *lacZ* amber mutations on the transcribed strand were recently isolated in approximately 10-fold excess over mutations on the non-transcribed strand upon treatment with the mutagen nitrous acid^3^. However, the prevalence of this molecular mechanism has not been studied in a non-mutator background and has not been validated in a whole genome context.

**Figure 1:**
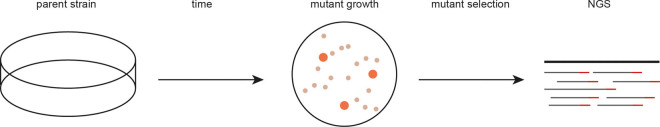
Schematic illustration of the experimental set-up. The parental strain is incapable of using the carbon source available in the plates, but after some days on plates, mutants adapted to their environment appear. These mutants are collected and subjected to next-generation genome sequencing (NGS).

To gain new, in-depth knowledge of the mechanisms behind adaptive mutation we studied the genetic changes in a background as undisturbed as possible. For this purpose, we designed an *E. coli* strain incapable of fermenting maltose, plated it on rich medium with maltose, and subsequently collected all mutants starting to outgrow on colonies over the course of two months (Fig. 1).

## Results

### Ageing colonies give rise to mutants in a non-mutator-based experimental system

Our starter strain is a derivative of the model bacterium *Escherichia coli* K12 MG1655 with its *cyaA* gene deleted, precluding synthesis of the signalling molecule cyclic AMP. As a consequence, a great many genes involved in carbon source catabolism cannot be expressed because they belong to operons requiring the cAMP Receptor Protein (CRP) complex to be activated^4^. These cells can grow for some time on rich media, but, after having used the accessible sources present in the available medium (maltose-MacConkey), the cells cannot grow further but remain as small colonies caught in a quiescent state.

**Figure 2:**
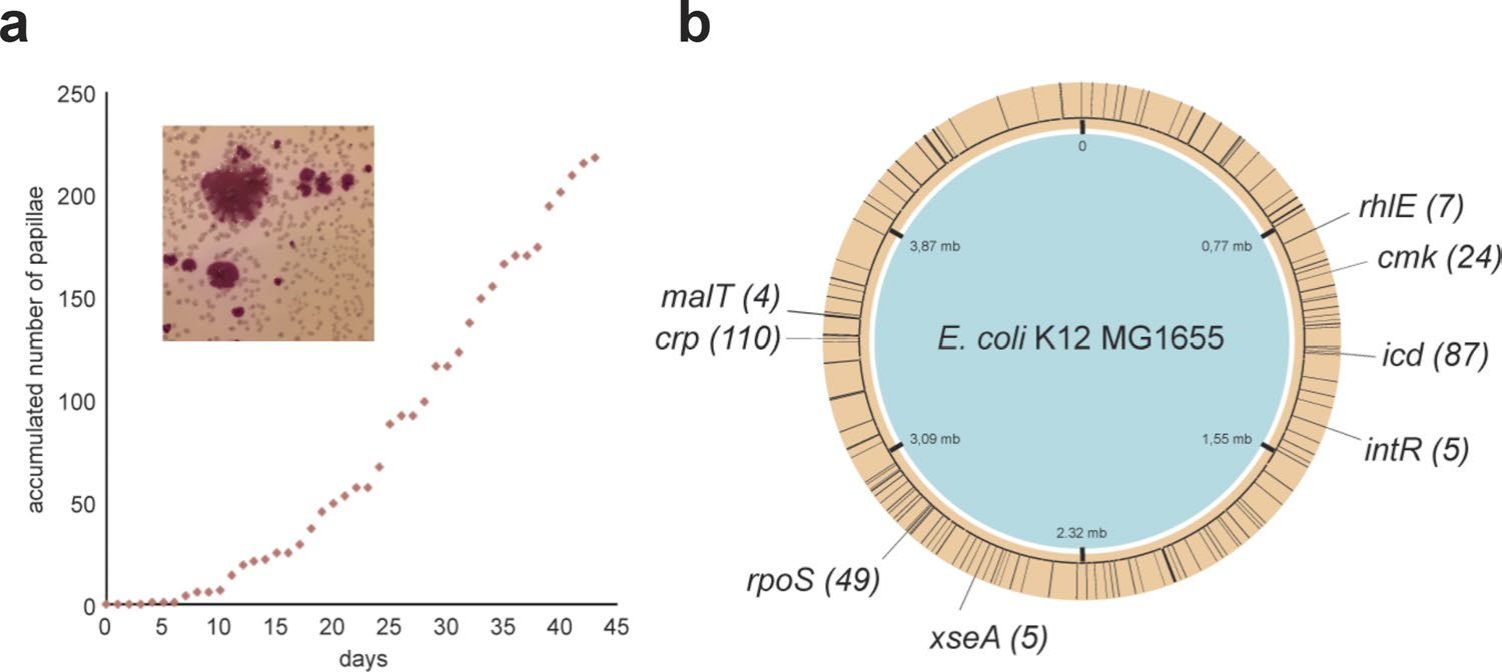
When subjected to prolonged incubation, *Escherichia coli* K12 MG1655 *cyaA*- cells adapt to their environment. (**a**) *E. coli cyaA-* cease to grow after forming small white colonies, when maltose is the major carbon source. After 4–5 days of incubation, mutants start occurring as red papillae on MacConkey agar (inserted image) and continue to form over approximately 6 weeks. Mutant papillae outgrew on approximately one in 200 colonies. (**b**) 96 mutant papillae were isolated, phenotypically characterized and genome sequenced. Loci with less than three mutations are uniformly distributed on the circular genome as illustrated with black lines. Hotspots of genes with more than two mutations are indicated with double-sized black lines and the total number of mutations in each gene is stated within brackets.

Around four days after plating, mutants capable of fermenting maltose started to appear as red papillae-like structures on the white colonies, and these mutants continued to appear over the next two months as some cells adapted to their environment. Mutant papillae outgrew on approximately one in 200 colonies, and progressively invaded the surface of the plate (Fig. 2a) making it necessary to start with a large number of plates to be able to sample mutants at the late time points. All mutant strains were purified. They displayed a variety of phenotypes on different carbon sources (Supplementary Table 1).

### Adaptive mutations are located in specific hotspot genes

We sequenced the genomes of 96 mutants, spanning the whole two-month period, and identified an average of four to five mutations per genome, the majority localized in a few hotspots only (Fig. 2b and Supplementary Table 2). The hotspots are located in the following genes (Fig. 2b): *cmk* (cytidylate kinase), *crp* (cyclic AMP receptor protein), *malT* (transcription activator of the maltose regulon transcription), *rhlE* (ATP-dependent DNA helicase), *rpoS* (sigma factor sigma 38), *xseA* (large subunit of exonuclease VII), and two loci that are known to be unstable in laboratory strains; *e14-icd* (e14 prophage inserted in the NADP-dependent isocitrate dehydrogenase gene^5^) and *intR* (Rac prophage integrase^6^).

From the way the experiment was constructed, it could be expected that mutations in the *crp* gene would account for growth on maltose in a *ΔcyaA* background. However, the difficulty of obtaining cAMP-independent (crp*) mutants witnessed by Jon Beckwith’s laboratory suggested that under exponential growth such mutations were very rare (of the order of 1 in 10^9^ cells^7^). In light of this, the ease with which we obtained such mutations in resting colonies was utterly unexpected.

It has been repeatedly observed that in *E. coli* laboratory strains, the *rpoS* gene is often inactivated^8, 9^. This is also what we observed at the *rpoS* hotspot (point mutations in general, but also frameshifts and a deletion of the region, see Supplementary Table 2). Inactivation of the RpoS protein may have triggered an increase in age-related mutagenesis, as this transcription factor is involved in oxidative stress response during stationary phase^10^. However, before we understand the molecular mechanism, we can only speculate why the remaining hotspot genes are targeted.

### The majority of adaptive mutations take place on the transcribed strand

**Figure 3:**
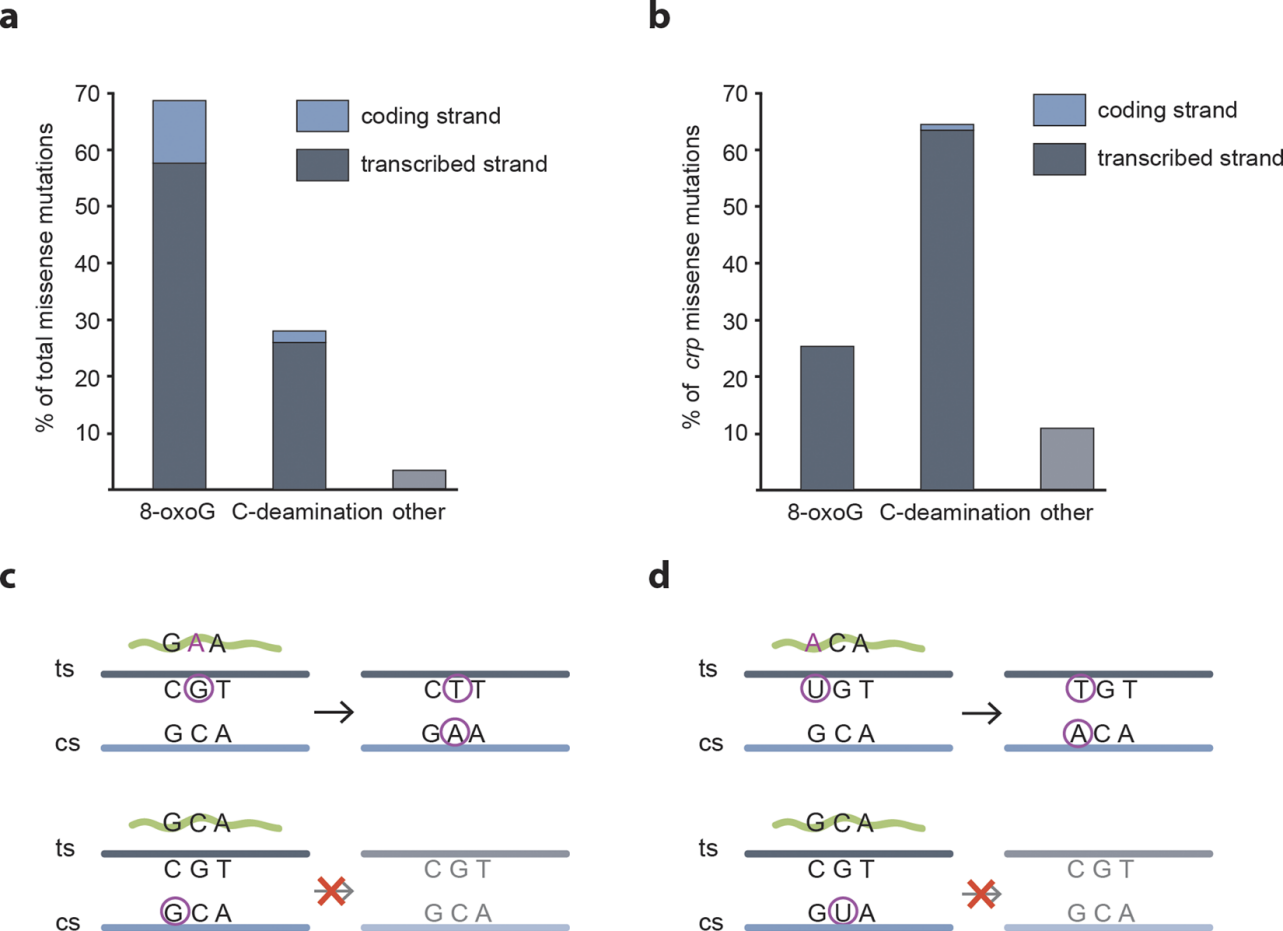
8-oxoguanine formation and cytosine deamination are highly dominating on the transcribed strand in isolated papillae. (**a**) Assuming that all identified G-T and C-A transversions are the result of 8-oxoguanine formation, and all G-C to A-T transitions are the result of cytosine deamination, we analysed the localisation of the corresponding G- and C- residues in the 96 sequenced mutant genomes and in (**b**) the 594 sequenced *crp* sequences. Only missense mutations were analysed. (**c**) Upper panel: Illustration of the mechanism of retromutagenesis after 8-oxoguanine (encircled G) formation on the transcribed strand (ts, dark grey line) that enables insertion of an adenine ribonucleotide in the mRNA (green waved line). The mutated mRNA enables growth and a round of replication that permanently fixes the mutation on the coding strand (cs, light blue line) in daughter cells. Lower panel: 8-oxoguanine formation on the coding strand does not transfer to daughter cells. (**d**) Illustration of retromutagenesis after cytosine nucleotide deamination into uracil (encircled U), enabling base pairing with A. Upper and lower panels show result of cytosine deamination on transcribed and coding strand respectively, as detailed in (**c**).

Theoretically, 12 different types of mutations are possible in DNA (A-C/G/T, C-A/G/T, G-A/C/T or T-A/C/G), but it is established that, due to respiration under stationary phase conditions, 8-oxo-G induced mutations are the most frequent to occur in the absence of an additional mutagenic process^11, 12^. In line with this, a dominant proportion of the mutations that were observed during the generation of the carbon-positive papillae is consistent with the involvement of 8-oxo-G (G-T and C-A transversions, 69% of the total number of missense mutations, Fig. 3a). A large proportion of the remaining mutations (28% of the total number of missense mutations identified) are likely the result of cytosine deamination (G-C to A-T transitions, Fig. 3a), another common mutational event in non-dividing cells^11, 13^.

Remarkably, the mutated base is highly dominantly present on the transcribed strand within gene coding sequences (84% for G transversions and 93% for C deaminations, Fig. 3a). Even more extremely, when counting in *crp* variants we Sanger sequenced from 594 different papillae (Supplementary Table 3), 99% of the G transversion and C deamination events had taken place on the transcribed strand (Fig. 3b). These observations are consistent with an increased mutation rate in transcribed regions (see Supplementary Table 2) and strongly support the retromutagenesis model (Fig. 3c,d)^1, 3, 14^ – here in the absence of a mutagen. Importantly, the extreme strand bias considerably reduces the relevance of potential residual replication and growth in the aging colonies – a heavily debated, but hard to test, part of the controversy of adaptive mutations^15^.

In the retromutagenesis model, mutations generated on the transcribed strand are selected for. In contrast, a so-called hitchhiking mutation is per definition not selected. Are the other hotspots selected for or hitchhikers? It is interesting to compare the specific nucleotide changes in *crp* that occur alone (singles) with those found only in combination (paired) with a crp* mutation: 92% (486 out of 528) of single mutations were G transversion and C deamination events on the transcribed strand, whereas only 59% (48 out of 81) of the paired mutations were of this type (Supplementary Table 3). Furthermore, the 1% G transversions and C transitions found on the non-transcribed strand (seven events in total) were all in the paired positions. Thus, in contrast to the crp* mutations, it is not possible to decipher the mechanism leading to these extra mutations in *crp*.

C deamination events outside *crp* are evenly distributed (50% on the transcribed and the non-transcribed strand). This indicates that the other C deamination events are not caused by retromutagenesis. Apart from events in *crp*, G transversion mutations were identified in the *xseA*, *cmk*, *malT* and *rpoS* hotspots, and 20 out of 21 *cmk* and 5 out of 5 *xseA* G transversions had taken place on the transcribed strand. This may indicate a selective advantage of these mutants, but hitchhiking is still theoretically possible because the *xseA* and *cmk* genes are transcribed from the same (+) strand as *crp* on the genome and thus may be fixed in the same round of replication as the crp* mutants are. In contrast, *rpoS* mutations must have a selective advantage: 16 out of 17 *rpoS* G transversion missense mutations occur on the transcribed strand, but the *rpoS* gene is placed on the complement (−) strand and thus retromutagenesis must have taken place independently of crp* generation. Three out of four identified G transversion mutations in the *malT* hotspot were placed on the non-transcribed strand and thus cannot have been selected by retromutagenesis.

### Phenotypes of selected mutations created by recombineering

Because selection is an important aspect of retromutagenesis^3^ it was interesting to study the phenotypes of the hotspot mutations in isolation and in different combinations. To this end, using oligonucleotide-based recombineering^16^, we re-created the most frequently occurring *crp* (A145T and A145E), *rpoS* (N98K), *cmk* (A216E) and *xseA* (H456N) mutations; as well as the *crp* (A145E) *rpos* (N98K), *crp* (A145T) *rpos* (N98K), *crp* (A145T) *cmk* (A216E) and *crp* (A145T) *xseA* (H456N) double mutants. The two *crp* mutants showed a crp* phenotype (red on mannitol-MacConkey) in isolation and in all combinations, but were not sufficient to enable fermentation of maltose (Table 1). In contrast, mutations in the hotspots genes *rpoS* or *xseA* showed no positive impact on the fermentation of different carbon sources tested except for a slightly increased sorbitol fermentation when combining the *rpoS* mutation with one of the *crp* mutations. The *crp* (A145T) *cmk* (A216E) double mutant was able to ferment both maltose and the other tested carbon sources - in agreement with the observation that the majority of *cmk* mutants (23/24) isolated in the papillae experiment all co-occurred with *crp* mutations and exhibited a red phenotype on maltose-MacConkey. Curiously, we were unable to isolate the *cmk* (A216) version alone as it always co-occurred with a (spontaneous) *crp* mutation – possibly indicating a strongly increased selection pressure for creating a *crp* mutant in the presence of this *cmk* mutation.

**Table 1:** Phenotypic characterisation of selected mutants created by recombineering.

Strain	Maltose	Mannitol	Glycerol	Sorbitol
*wt* (*cyaA*::*cat Δfnr)*	white	white	white	white
*crp* A145T	white	red	fisheye	white
*crp* A145E	white	red	white	white
*rpoS* N98K	white	white	white	white
*xseA* H456N	white	white	white	white
*crp* A145T *rpoS* N98K	white	red	fisheye	fisheye
*crp* A145E *rpoS* N98K	white	red	fisheye	fisheye
*crp* A145T *xseA* H456N	white	red	fisheye	white
*crp* A145T* *cmk* A216E	red	red	fisheye	fisheye

The phenotypes of the engineered *E. coli* strains were assayed on MacConkey medium supplemented with maltose, mannitol, glycerol or sorbitol.

^*^The *cmk* A216E mutant was genome sequenced and contained three additional missense mutations *gss* A240D, *gspC* V256I and *malT* G60S.

### Formation of adaptive mutations shows age-dependence

**Figure 4:**
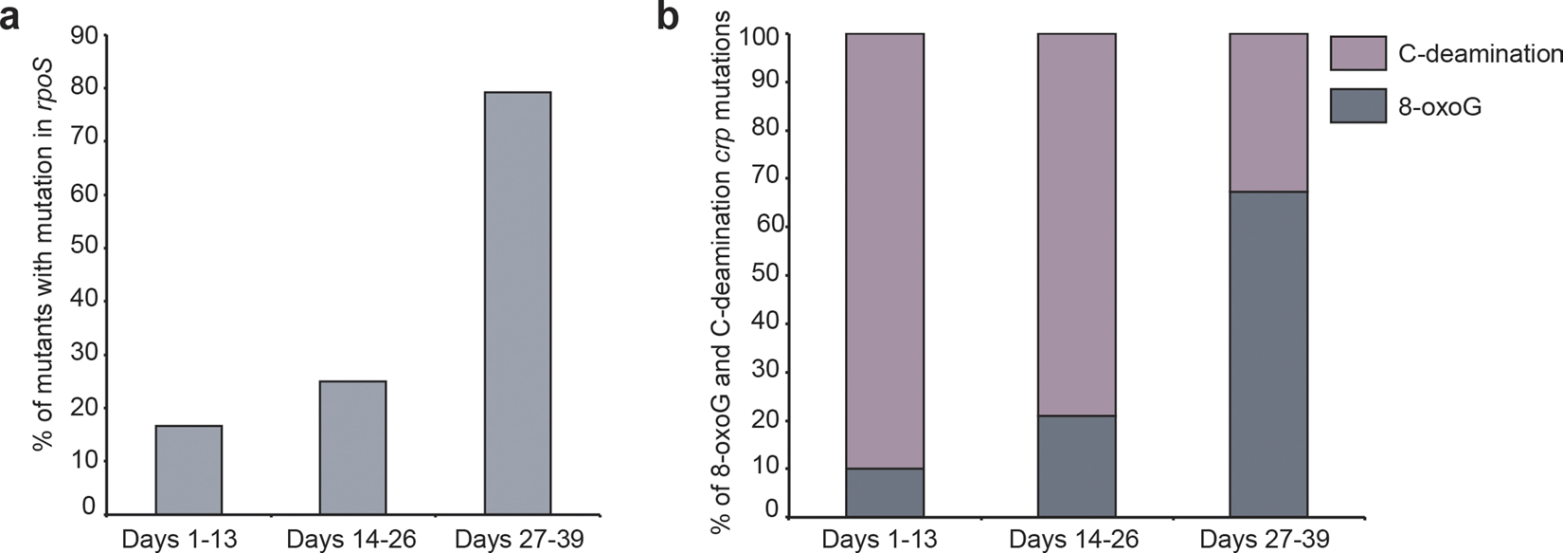
Time-dependent trends in mutational mechanisms. (**a**) The frequency of *rpoS* mutations in isolated mutants is increasing with time. The x-axis shows days after plating. (**b**) Isolated mutants show a distinct mutation pattern in the *crp* gene. Over time, there is a steady increase in mutations originating from guanosine (likely 8-oxo-G) whereas the C-deamination associated mutations decrease.

Time is an important factor in the development of mutant papillae and the sampling of adaptive mutants over two months enabled us to observe interesting age-related trends. Firstly, a minimum of four days of incubation is required for adaptive mutations to occur in our experimental setup. Secondly, the frequency of papillae with *rpoS* mutations increases over time, suggesting an increasingly selective advantage with age (Fig. 4a). Alternatively, the mechanism(s) leading to *rpoS* mutations are becoming more prominent over time. Thirdly, G transversion mutations in *crp* increases over time, paralleled by a decrease in C-deaminations (Fig. 4b).

Our observations are not consistent with a general increase in mutagenesis, as the frequency of rifampicin resistance is of the order of four mutants per 10^8^ cells as generally observed^17^. Furthermore, removal of RecA, a previously suggested key actor in adaptive mutations^18^, displayed only a marginally negative effect on the total number of papillae formed (Supplementary Figure 1).

## Discussion

In the present study, we sequenced 96 adaptive mutants isolated during a two-month period, after having arisen from a minimally perturbed genetic background. To our knowledge, a similar experiment has not been performed at this time scale previously.

Within the paradigm of our selection procedure, the identification of mutations in genes *crp* and *rpoS* was not unexpected. CRP is known to control carbon catabolism and Sabouring and Beckwith isolated the first CRP* mutant in a *cya* background as a strain growing on minimum maltose plates^7^. In the same way, RpoS controls expression of genes important for stationary phase survival. Furthermore mutations in the cognate genes have been repeatedly identified in carbon-limitation evolution experiments^19–21^. Remarkably, besides genes relevant to the process of carbon source usage, the sequencing of 96 papillae genomes revealed several hotspots and several regions of instability. The latter are all related to mobile elements. In particular the *icd* gene is the place of prophage e14 attachment site^5^, and it appears from our data that this region remains unstable in our experimental conditions. A similar observation was made when investigators explored the GASP phenotype^22^. In fact, the whole region is unstable as witnessed by mutations in genes *ymfE*, *ymfI* and *ymfJ* (see Supplementary Table 2). In addition to mutations in *crp*, *rpoS* and *malT*, gene *cmk*, coding for cytidylate kinase, emerged as a mutational hotspot. In the *cmk rpsA* operon^23^, the latter gene coding for ribosomal protein S1 in Proteobacteria and an mRNA binding protein in Firmicutes, is widely conserved in Bacteria^24^. Cytidylate kinase is critical for the production of CDP, the immediate precursor of dCDP that is an absolute requirement for the presence of cytosine in DNA^25^, and a major source of thymine as well^26^. The metabolism during stationary phase may require maintenance of a stable CTP pool, asking for the presence of both CMK and nucleoside diphosphokinase, but the reason for an increased mutation number in the *cmk* gene is unknown. However, an unexpected phenotype of the *cmk* gene revealed that it was involved in the control of chromosome maintenance (*cmk* was originally named *mssA*, suppressor of a cold sensitive growth of mutants in the uridylate kinase gene^27^), suggesting that it may belong to some important role in this domain.

The present work resulted in the selection of a very large number of CRP variants. It seems that despite the number of mutants explored (*ca.* 600), there is still room for further mutants to appear. The phenotype of the papillae was assayed on MacConkey medium supplemented with maltose, mannitol, glycerol or sorbitol, as well as on EMB maltose plates (Supplementary Table 1). The corresponding phenotypes are driven by the sequence of the CRP protein, but mutations elsewhere in the genome can also impact the phenotype (see for example in Supplementary Table 1 papilla 1 compared to papilla 3). The present work could be a first step in the understanding of functional networks directing carbon source utilization in stationary phase. Remarkably, previous knowledge was far from covering the whole set of what we observed. This highlights a rarely underlined process of evolution that can be explored using experiments similar to the one presented in this article.

The two maltose-positive clones that carried a wild-type *crp* had a weak positive phenotype and a mutation in *rpoS*, besides other mutations. These mutants, in contrast to the mutants in the *crp* gene, did not grow on minimal plates supplemented with maltose. They may have uncovered pathways allowing for growth on components of the rich MacConkey medium that the starter strain could not use.

Finally, noting that we did not find any hint of RecA involvement in the genomes we sequenced, and to validate a possible role of RecA in adaptive mutation as previously suggested^18^, we introduced a *recA* mutation in our root strain and monitored the appearance of positive papillae. Interestingly, while, overall, there does not seem to be a large decrease of the total number of papillae in a *recA*- background, the time course of their appearance is considerably retarded as compared to the situation observed in the *cyaA-* background (Supplementary Figure 1). Indeed, it takes a long time for the first papillae to appear in the *recA-* background, but their number keeps increasing, for up to two months, with the total number observed similar to that found in the *cyaA-* reference strain. In our context, *recA* mutants are growing more slowly than their parent counterpart. Whether this growth rate can account for the delay in papillae appearance warrants further studies.

Age was an essential factor not only for the development of adaptive mutation, but also seemingly for the mutational mechanism and we show that a highly dominating proportion of the adaptive mutations originate from guanosine on the transcribed strand - likely due to 8-oxo-G modification. This strongly supports the retromutagenesis model as a major mechanism behind adaptive mutations. Were this process generalised to multicellular organisms it would be a fertile contribution to the initiation of cancer that parallels ageing.

## Methods

### Bacterial strains

Because the *fnr* gene is a homologue of *crp*, we used a starter strain previously identified as MG1655 at the ECGSC1 shown to harbour a ca. 40 kb deletion in the *fnr* region^28^. The *cyaA* deletion was introduced with lambda Red-induced recombination^29^. In pilot experiments, we had noticed that the presence of a pTrc99A plasmid (Pharmacia Biotech) carrying the *E. coli tig* gene (PCR amplified with the oligonucleotides CATGCCATGGTGAGGTAACAAGATGCAAGTTTC 3′ and 5′ CGCGGATCCAATTACGCCTGCTGGTTCATC 3′ and cloned into the *Nco*I and *Bam*HI restriction sites) produced twice as many mutants that in absence of the *tig* plasmid, typically allowing us to recover around 200 mutants in each two months experiment, a figure quite convenient to get significant observations. Thus K12 Mg1655 (*cyaA*::*cat* delta *fnr*, pTrc*tig*) is our starter strain. The *recA* deletion was from the Keio collections^30^ that was used to prepare a P1 lysate by standard procedures.

### Papillae formation assay

For growth on plates the lactose-free MacConkey medium was used^31^ (Difco MacConkey Agar Base) supplemented with 1% carbohydrate (maltose in most experiments) or glycerol and chloramphenicol, 5 mg/litre, ampicillin, 100 mg/litre, and 1 mM IPTG. For subsequent 3-times purification of papillae, chloramphenicol, ampicillin and IPTG were omitted from the plates. To obtain isolated colonies on the plates, early stationary-phase grown bacteria from the LB liquid culture (37 °C) were diluted in sterile water containing 9 g/l sodium chloride, to the concentration of 2.5 × 10^4^ bacteria/ml and 100 µl of the bacterial suspension was spread onto MacConkey plates. The plates were subsequently placed in plastic boxes containing beakers with water to ensure constant humidity and placed for the duration of the experiment in an incubator at 37 °C. To test for mutator phenotypes, rifampicin agar plates were prepared using the LB medium agar supplemented with 100 mg/litre rifampicin prepared in methanol. At all times the plates were wrapped in aluminium foil for protection from light.

### Genome sequencing

The genomic libraries were generated using the TruSeq®Nano DNA LT Sample Preparation Kit (Illumina Inc.). 498 independent papillae were selected for amplification of the *crp* region by direct colony PCR. The PCR reaction was made using Red-Taq polymerase (Sigma-Aldrich) according to the manufacturer’s instructions (hybridisation at 55 °C) with the following primers: forward primer 5′TTTCGGCAATCCAGAGACAGC3′ and reverse primer 5′AACATAGCACCAGCGTTTGTCG3′. The amplified *crp* regions were sequenced by Sanger method with two primers: forward 5′TTATCTGGCTCTGGAGAAAGC3′ and reverse primer 5′TCGAAGTGCATAGTTGATATCGG3′.

### Genome engineering

K12 Mg1655 (*cyaA*::*cat* delta *fnr*) was transformed with plasmid pMA7^16^ and recombineering performed using the oligonucleotides 1–5 (Supplementary Methods) was carried out as described by Sandberg *et al*.^32^ with three exceptions: (1) overnight LB liquid cultures were supplemented with 0.4% glucose to preclude starvation, (2) 1 mM cAMP (Sigma-Aldrich A9501) was supplemented at time of induction, and (3) cells were spread on MacConkey-mannitol plates (1% mannitol, 5 ug/ml chloramphenicol, 50 ug/ml ampicillin). Two rounds of recombineering were applied for *crp* A145T, *crp* A145E, and *xseA* H456N and four rounds for *cmk* A216E and *rpoS* N98K. Colonies were screened for the desired point mutations by sequencing. Positive clones were either streaked repeatedly on MacConkey-mannitol plates without ampicillin until the pMA7 plasmid was lost or inoculated in liquid culture to carry out recombineering of new targets as above (2 rounds for both *crp* A145E into *rpoS* N98K background and for *crp* A145T into *xseA* H456N background). The MacConkey medium was applied to ensure that only the dominant phenotype was propagated into later experiments and thus to prevent development of and contamination by unwanted mutations in the background. The *crp* A145T mutations occurred spontaneously in *rpoS* N98K cells and in *cmk* A216E. We were unable to obtain the *cmk* A216E background without spontaneous *crp* and other mutations.

## Electronic supplementary material

Supplementary Information

Supplementary Information

Supplementary Information

Supplementary Information
