# Supplementary material for: Generation of mutation hotspots in ageing bacterial colonies
Source: Sci Rep. 2016 Dec 5;6:2. doi: 10.1038/s41598-016-0005-4 (PMC5431349; doi:10.1038/s41598-016-0005-4)
Supplement: Supplementary file 1 — Supplementary Information [file 41598_2016_5_MOESM1_ESM.doc]

**Supplementary information to**

**Generation of mutation hotspots in ageing bacterial colonies**

Agnieszka Sekowska, Sofie Wendel, Emil C. Fischer, Morten H. H. Nørholm and Antoine Danchin

**Supplementary methods**

*Escherichia coli* K12 remains the best-known living species. The genomes of several laboratory strains have been sequenced, and it has been observed that there is significant variation between strains ^1^. Two major sources of laboratory strains have been investigated, MG1655, by Fred Blattner and co-workers ^2^, and W3110 by Hirotada Mori and co-workers (origin of the Keio collection ^3^). Strain MG1655 seems to be the closest isolate from the original K12 isolate, which has now been lost. Beside a range of variants present in different laboratories, an initial isolate of MG1655 deposited at the *Escherichia coli* Genetic Stock Center (ECGSC) was shown to harbour a ca. 40 kb deletion in the *fnr* region ^1^. For our experimental chassis we wished to construct a *cyaA* deletion strain, preventing cells from synthesising cyclic AMP. We expected that among adaptive mutants we might obtain *crp* derivatives of the *crp* gene (*crp**), coding for variants of the Cyclic AMP Receptor Protein (CRP), as previously obtained by Sabourin and Beckwith ^4^. Because the FNR protein is strongly similar to CRP we were concerned that we might stumble on interference between *crp* and *fnr* that would have been confounding our observations. For this reason we decided to use as a chassis the strain previously identified as MG1655 at the ECGSC ^1^, a strain that was used with success recently in experiments exploring the swarming behaviour of *E. coli* ^5^. In preliminary experiments we indeed found that papillae were produced on our selective medium (MacConkey plates supplemented with maltose as the superfluous carbon source). However, we wished to make the most of our experimental set-up, while we had noticed that many *E. coli* strains, when streaked on plates for conservation and then reused, had lost the activity of their *rpoS* gene ^6–8^. RpoS has an important role under stationary phase conditions, in particular because it controls synthesis of a protein that should have an important role in our experiment, the ribosome trigger factor (TIG), involved in the folding of proteins *in statu nascendi* ^9^. We therefore introduced in our chassis, beside a replacement of the *cyaA* gene by a chloramphenicol cassette, a plasmid coding for *tig*. We noticed that the number of papillae produced under such conditions was about twice that in absence of the *tig* plasmid, allowing us to recover some 100 papillae or so in each two months experiment, a figure quite convenient to get significant observations. Our chassis is therefore strain AMBEC7001 (*cyaA*::*cat* *Δfnr*, pTrc*tig*).

In our set-up we expected that *crp** mutations would display a GASP phenotype. However, the experiments by Jon Beckwith's laboratory ^4^ showed that under exponential growth constitutively positive *crp* mutations were very rare (of the order of 1 in 10^9^ cells). Mutagenesis would considerably increase the amount of such mutations. Hence, inactivating antimutator genes would result in an overall increase of mutations, without allowing much insight into the process. For this reason we were careful to avoid bacterial chasses that carry mutator genes. In particular, the MutM and MutY proteins correct mutations induced by the presence of 8-oxoguanine ^10,11^; MutM removes 8-oxoG paired with C in DNA whereas MutY removes A paired with 8-oxoG in *syn* conformation in the double helix. As an essential requirement, the *mutM* and *mutY* genes are undamaged in our chassis. Indeed, our preliminary experiments showed that the strains submitted to ageing in stationary phase did not display an increase in rifampicin resistant mutants (*rpoB*).

The genomic libraries were generated using the TruSeq®Nano DNA LT Sample Preparation Kit (Illumina Inc.). Briefly, 100 ng of genomic DNA was diluted in 52.5 µl TE buffer and fragmented in Covaris Crimp Cap microtubes on a Covaris E220 ultrasonicator (Woburn). According to Illumina’s recommendations for a 350-bp average fragment size, the settings used were 5% duty factor, 175 W peak incident power, 200 cycles/burst, and 50-s duration under frequency sweeping mode at 5.5 to 6°C. The ends of fragmented DNA were repaired by T4 DNA polymerase, Klenow DNA polymerase, and T4 polynucleotide kinase. The Klenow exo minus enzyme was then used to tail an 'A' base to the 3’ end of the DNA fragments. After ligation of adapters, DNA fragments ranging from 300 - 400 bp were recovered by bead purification. Finally, the adapter-modified DNA fragments were enriched by 3 cycles of PCR. The final concentration of each library was measured by Qubit® 2.0 Fluorometer and Qubit DNA Broad range assay (Life Technologies). Average dsDNA library sizes were determined using the Agilent DNA 7500 kit on an Agilent 2100 Bioanalyzer. Libraries were normalised and pooled in 10 mM Tris-HCl, pH 8.0, 0.05% Tween 20 to a final concentration of 10 nM. 10 pm pools of 20 libraries in 600 µl ice-cold HT1 buffer were denatured in 0.2N NaOH, loaded onto the flow cell provided in the MiSeq Reagent kit v2 and sequenced on a MiSeq (Illumina Inc.) platform, with a paired-end protocol and read lengths of 151 nt. The Illumina sequencing data was quality-trimmed using the Trimmomatic tool (version 0.32) ^12^ with the settings CROP:145 HEADCROP:15 SLIDINGWINDOW:4:15 MINLEN:30. The cleaned data was used as input for variant calling using the breseq pipeline (version 0.26.0) ^13^ with –j 4 and –b 20 as the only changes to default settings. The reference strain for this analysis was *Escherichia coli* MG1655 with the accession number NC_000913.3.

Recombineering was performed with the oligonucleotides:

(1) *crp* A145T: 5’-GTGACGGGCCGCATTaCACAGACTCTGCTGAATCTGGCAAAACAACCAGACGCTATGACTCACCCGGACGGTATGCAAATCAAAATTACC-3’; (2) *crp* A145E: 5’-GTGACGGGCCGCATTGaACAGACTCTGCTGAATCTGGCAAAACAACCAGACGCTATGACTCACCCGGACGGTATGCAAATCAAAATTACC-3’ (3) rpoS N98K: 5’-ACCACGATTGCCATAACGGCGGGCAATTTTTACCACCAGACGCAAtTTACTCTCGATCATCCGGCGGCGAGAGGCGACATCTCCACGCAG-3’; (4) cmk A216E: 5’-CGGTCGCTTATGCGAGAGCCAATTTCTGGCGCGCGTATTGTAGCtCTTTTTCAATCACTTGCTCAATGCTTAAGGTGGTGGAATCCAACA-3’; and (5) xseA H456N: 5’-GAAGTAAAAAACATCCAGCCAGTAAAAAAATCGCGTAAAAAGGTGaATTAAGCCAGCACGAATTCAACGCGTTTTTTCGATATCAAGCCA-3’

**Supplementary figure**


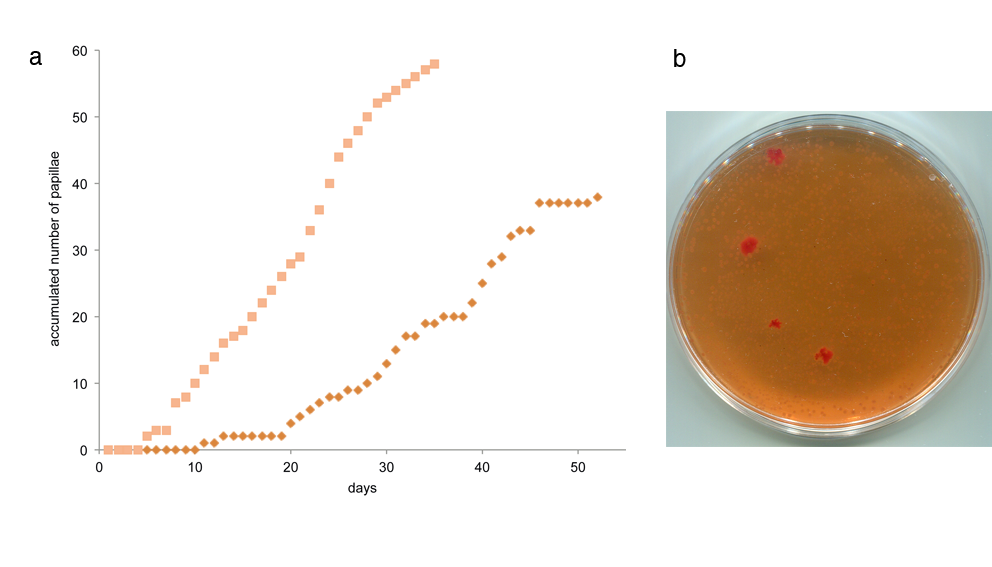


**Supplementary Figure 1. Time course of papillae formation in a *cyaA-* strain and *cyaA* *recA* double mutant**. **(a)** Papillae formation is delayed in the *cyaA recA* (orange diamonds) mutant compared to the *cyaA* deficient reference strain (light orange squares). In the *cyaA* mutant the first papillae are appearing on the 5^th^ day of experiment and terminating over the 32^nd^ day, while in the *cyaA recA* double mutant the first papillae are appearing on the 10^th^ day and keep appearing on the 52^nd^ day after the inoculation. Papillae are represented as accumulated total number of occurrences. **(b)** An example of a typical plate with the papillae after 6 days.

**References to supplementary methods**

1. Soupene, E. *et al.* Physiological studies of *Escherichia coli* strain MG1655: growth defects and apparent cross-regulation of gene expression. *J Bacteriol* **185,** 5611–5626 (2003).

2. Blattner, F. R. *et al.* The complete genome sequence of *Escherichia coli* K-12. *Science (80-. ).* **277,** 1453–1462 (1997).

3. Hayashi, K. *et al.* Highly accurate genome sequences of *Escherichia coli* K-12 strains MG1655 and W3110. *Mol Syst Biol* **2,** 2006 0007 (2006).

4. Sabourin, D. & Beckwith, J. Deletion of the Escherichia coli crp gene. *J Bacteriol* **122,** 338–340 (1975).

5. Liu, C. *et al.* Sequential Establishment of Stripe Patterns in an Expanding Cell Population. *Science.* **334,** 238–241 (2011).

6. Dong, T., Chiang, S. M., Joyce, C., Yu, R. & Schellhorn, H. E. Polymorphism and selection of *rpoS* in pathogenic *Escherichia coli*. *BMC Microbiol* **9,** 118 (2009).

7. Jishage, M. & Ishihama, A. Variation in RNA polymerase sigma subunit composition within different stocks of *Escherichia coli* W3110. *J Bacteriol* **179,** 959–963 (1997).

8. Visick, J. E., Cai, H. & Clarke, S. The L-isoaspartyl protein repair methyltransferase enhances survival of aging *Escherichia coli* subjected to secondary environmental stresses. *J Bacteriol* **180,** 2623–2629 (1998).

9. Silva, I. J., Ortega, A. D., Viegas, S. C., Garcia-Del Portillo, F. & Arraiano, C. M. An RpoS-dependent sRNA regulates the expression of a chaperone involved in protein folding. *RNA* **19,** 1253–1265 (2013).

10. Michaels, M. L., Cruz, C., Grollman, A. P. & Miller, J. H. Evidence that MutY and MutM combine to prevent mutations by an oxidatively damaged form of guanine in DNA. *Proc Natl Acad Sci U S A* **89,** 7022–7025 (1992).

11. Tajiri, T., Maki, H. & Sekiguchi, M. Functional cooperation of MutT, MutM and MutY proteins in preventing mutations caused by spontaneous oxidation of guanine nucleotide in *Escherichia coli*. *Mutat Res* **336,** 257–267 (1995).

12. Bolger, A. M., Lohse, M. & Usadel, B. Trimmomatic: a flexible trimmer for Illumina sequence data. *Bioinformatics* **30,** 2114–2120 (2014).

13. Deatherage, D. E. & Barrick, J. E. Identification of mutations in laboratory-evolved microbes from next-generation sequencing data using breseq. *Methods Mol Biol* **1151,** 165–188 (2014).

**Supplementary Tables**

**Supplementary Table 1. Phenotypic characterisation of the 96 isolated mutants.**

The phenotypes of the papillae (Pap) were assayed on MacConkey medium supplemented with maltose, mannitol, glycerol or sorbitol, as well as on EMB maltose plates. On MacConkey plates, colonies can be either red or white, and in some cases red at the centre but white around ("fisheye" phenotype). On EMB, colonies can be very dark (black), often with a remarkable green shine (gold green), purple, or in some cases white.

**Supplementary Table 2. Mutations in the 96 genomes sequenced using NGS.**

The mutation sites observed in the 96 mutants are described. The origin of the mutation is colour coded: G->T transversions are in blue while C->A transversions are in green; C->T transitions are in yellow and G->A transitions are in pink. Hotspot regions are coloured light grey (and the *crp* region is in orange).

**Supplementary Table 3. CRP mutations in a total of 594 additional papillae.**
